# Physical exertion as a risk factor for perimesencephalic nonaneurysmal subarachnoid hemorrhage

**DOI:** 10.1002/brb3.2756

**Published:** 2022-09-01

**Authors:** Dan Laukka, Juri Kivelev, Riitta Rautio, Johanna Kuhmonen, Matias Sinisalo, Jaakko Rinne, Melissa Rahi

**Affiliations:** ^1^ Department of Neurosurgery, Neurocenter Turku University Hospital Turku Finland; ^2^ Department of Radiology Turku University Hospital, University of Turku Turku Finland; ^3^ Neurocenter Turku University Hospital Turku Finland; ^4^ Clinical Neurosciences University of Turku Turku Finland

**Keywords:** nonaneurysmal subarachnoid hemorrhage, perimesencephalic subarachnoid hemorrhage, physical exertion, risk factors

## Abstract

**Background:**

Perimesencephalic and nonperimesencephalic nonaneurysmal subarachnoid hemorrhage (PM‐naSAH and NPM‐naSAH) have a different bleeding pattern and clinical course. The etiology and risk factors for PM‐naSAH and NPM‐naSAH are unclear. The objective of this study was to compare risk factors and triggering events between PM‐naSAH and NPM‐naSAH.

**Methods:**

We reviewed retrospectively all patients (*n* = 3475) who had undergone cerebral digital subtraction angiography between 2003 and 2020 at our tertiary hospital. Of these, 119 patients had 6‐vessel angiography negative subarachnoid hemorrhage (47 (39%) PM‐naSAH and 72 (61%) NPM‐naSAH) and accurate information about the triggering event was available in 42 (89%) PM‐NASAH and 64 (89%) NPM‐naSAH patients.

**Results:**

PM‐naSAH were younger compared to NPM‐naSAH (mean age [SD]; 55.3 [11.1] years vs. 59.6 [12.2] years, *p* = .045. PM‐naSAH was triggered during the physical exertion in 79% of patients and 16% of patients with NPM‐naSAH (relative risk 5.4; 95% CI, 2.9‐10.1, *p* < .0001). There were no significant difference in sex, smoking, alcohol abuse, hypertension, diabetes, hyperlipidemia, or anticoagulation/antithrombotic usage between PM‐naSAH and NMP‐naSAH, *p* > .05.

**Conclusion:**

Physical exertion was a triggering factor in most of the PM‐naSAH cases and the risk was five times greater than in NMP‐naSAH. More studies are needed to confirm our results and to study pathophysiology of PM‐naSAH and NPM‐naSAH.

## 1 INTRODUCTION

Subarachnoid hemorrhage (SAH) without vascular lesions on four vessel angiography is called nonaneurysmal subarachnoid hemorrhage (naSAH) and accounts around 15% of all SAH cases (Rinkel et al., 1991). Based on the bleeding pattern, naSAH can be divided in two subtypes, perimesencephalic naSAH (PM‐naSAH) and nonperimesencephalic nonaneurysmal naSAH (NPM‐naSAH) (Cánovas et al., 2012; Kang et al., 2009; Konczalla et al., 2016; Raya et al., 2014). NPM‐naSAH have more complications and worse outcome than PM‐naSAH (Cánovas et al., 2012; Kang et al., 2009; Konczalla et al., 2016; Raya et al., 2014), which might suggest different pathophysiological background.

Etiology and risk factors for naSAH is unclear and there are lack of studies comparing risk factors between PM‐ and NPM‐naSAH (Mensing et al., 2018). Physical exertion as a triggering event for aneurysmal SAH has been studied earlier (Anderson et al., 2003; Fann et al., 2000; Schievink et al., 1989; Vlak et al., 2011), but only a few case series have focused on association between physical exertion and nonaneurysmal SAH (Linn et al., 1998; Matsuyama et al., 2006).

The purpose of this study was to compare risk factors between PM‐naSAH and NPM‐naSAH, including physical exertion at the onset of SAH symptoms.

## 2 METHODS

This study was approved by the Southwest Finland hospital district's ethical committee and institutional review board. Research number ID is T110/2018 and number for approval decision is T04/005/18. Patient consent was waived based on the retrospective registry design.

We reviewed retrospectively all patients who underwent cerebral digital subtraction angiography (DSA) for any reason between 2003 and 2020 at our tertiary hospital (*n* = 3475). Our tertiary hospital is responsible of all nontraumatic subarachnoid hemorrhage patients in population of 890,000 citizens.

Patients who had undergone DSA for any reason other than subarachnoid hemorrhage were excluded based on a review of patient charts and radiographic findings (*n* = 2414). Those with initial negative CT scans for blood and in those where bleeding was restricted in cortical subarachnoid space (for example bleeding in one cortical sulcus) were excluded. DSA was done in 1061 patients because of SAH or suspicion of SAH. Of these, following patients were excluded: aneurysmal SAH (*n* = 906), no blood in initial CT‐scans, but suspicion of SAH based on cerebrospinal fluid examination or hemosiderosis in brain MRI (*n* = 17), traumatic SAH with a suspicion of secondary cause (*n* = 4), SAH restricted in the cortical subarachnoid space (*n* = 5), and SAH from arteriousvenous malformation or fistula (*n* = 10).

All together 119 patients had naSAH (72 NPM‐naSAH and 47 PM‐naSAH) bleeding pattern on initial CT scans and negative 6‐vessel cerebral DSA.

NaSAH was categorized in two types: (1) PM‐naSAH and (2) NPM‐naSAH based on the bleeding pattern. Following radiological criteria for PM‐naSAH was used based on bleeding pattern: (1) bleeding restricted on the pre‐pontine cistern, medial Sylvian fissure, and without complete filling of the anterior interhemispheric fissure and (2) no franc intraventricular bleeding (Rinkel et al., 1991). Bleeding extending into lateral Sylvian fissure, interhemispheric fissures, and/or ventricles were interpreted as NPM‐naSAH (Cánovas et al., 2012; Kang et al., 2009; Raya et al., 2014).

For PM‐naSAH and NPM‐naSAH, electronic patient records were reviewed for hypercholesterolemia, hypertension, diabetes mellitus type 1 and type 2, coronary artery disease, alcohol abuse, smoking status (smoker/ex‐smoker vs. never smoker), antithrombotic medication (aspirin, adenosine diphosphate receptor inhibitor and glycoprotein IIb/IIIa receptor inhibitors), and anticoagulation medication (warfarin, directly acting oral anticoagulants, heparin).

Similar to Schievink et al. (1989) study, triggering event for SAH was evaluated separately from the electronic patient records and if there was a clear mention in the records of what the patient was doing at the onset of the headache. Physical exertion was classified as an activity likely to involve Valsalva maneuver (defecation, heavy lifting, sports/exercise, sexual intercourse, sneezing/vigorous coughing, physical work, or housework). No physical exertion group included following activities: resting, sleeping, and light activity. Light activity was defined as an activity no heavier than normal walking.

### 2.1 Statistical analysis

All analyses were performed using SPSS Statistics 27(IBM Corp., Armonk, NY, USA).

Mean ages were compared with Mann–Whitney *U* test. Categorical variables were compared Fisher's exact test. Physical exertion was categorized as physical exertion versus no physical exertion and Fisher's exact test was used between group comparisons. *p* Values < .05 were considered statistically significant. Missing data was not included in the analysis.

## 3 RESULTS

**FIGURE 1:**
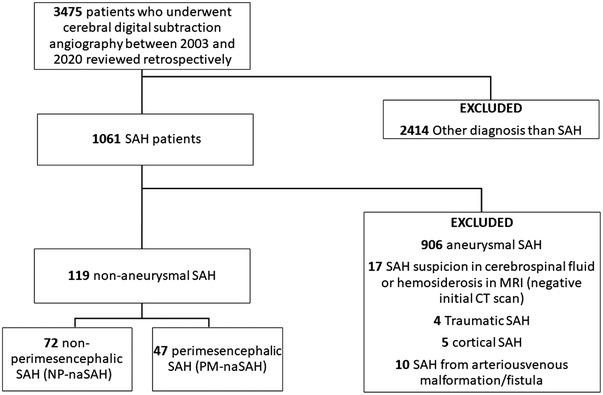
Flow chart of this study. SAH = subarachnoid hemorrhage. MRI = magnetic resonance imaging

Of the 1061 SAH patients with available cerebral DSA, 119 had nonaneurysmal SAH. From 119 nonaneurysmal SAH patients, 72 (61%) had NPM‐naSAH and 47 (39%) PM‐naSAH (Figure 1). Results and demographics are presented in Table 1. PM‐naSAH were younger compared to NPM‐naSAH (mean age [SD]; 55.3 [11.1] years vs. 59.6 [12.2] years, *p* = .045). There were no sex difference between PM‐naSAH and NPM‐naSAH (19 [40%] females vs. 36 [51%] females, *p* = .35). PM‐naSAH was triggered during the physical exertion in 79% of patients and 16% of patients with NPM‐naSAH (relative risk 5.4; 95% CI, 2.9–10.1, *p* < .0001). There were no significant difference in smoking, alcohol usage, hypertension, diabetes, hyperlipidemia, or anticoagulation usage (*p* > .05).

**TABLE 1:** Clinical characteristics of perimesencephalic nonaneurysmal subarachnoid hemorrhage (PM‐naSAH) patients and nonperimesencephalic nonaneurysmal subarachnoid hemorrhage (NMP‐naSAH) patients

Characteristic	PM‐naSAH (*n* = 47)	NMP‐naSAH (*n* = 72)	*p* Value
Age, years (SD)	55.3 (11.1)	59.6 (12.2)	.045
Female sex, *n* (%)	19 (40%)	36 (51%)	.35
Hypercholesterolemia, *n* (%)	10 (21%)	17 (24%)	.82
Hypertension	12 (25%)	25 (35%)	.31
Diabetes mellitus (type 1 or type 2)	6 (13%)	8 (11%)	1.0
Coronary artery disease	0 (0%)	3 (13%)	.54
Smoking/ex‐smoker^a^	13 (33%)	17 (29%)	.82
Alcohol abuse^b^	1 (3%)	4 (7%)	.63
Antithrombotic medication	1 (2%)	7 (10%)	.14
Anticoagulation medication	4 (9%)	11 (15%)	.27
Anticoagulation or antithrombotic medication	5 (11%)	18 (25%)	.06
Triggering event available, *n* (%)	42 (89%)	64 (89%)	
Physical exertion	33 (79%)	10 (16%)	<.0001
Sports/exercise	16 (39%)	5 (8%)	
Heavy lifting/heavy working	7 (15%)	2 (3%)	
Defecation	2 (5%)	2 (3%)	
Vigorous coughing or sneezing	5 (12%)	0 (0%)	
Sexual intercourse	1 (2%)	1 (2%)	
Housework	2 (6%)	0 (0%)	
No physical exertion	9 (21%)	54 (84%)	<.0001
Light activity	2 (5%)	20 (31%)	
Sitting/resting	7 (17%)	24 (38%)	
Sleeping	0 (0%)	10 (16%)	

^a^ Missing information of smoking history in 21 patients.

^b^ Missing information of alcohol usage in 29 patients.

## 4 DISCUSSION

We found that physical exertion is a triggering factor in most of the PM‐naSAH (79% of patients) and the risk of PM‐naSAH during the physical exertion was 5‐times higher than in NPM‐naSAH.

Risk factors for PM‐naSAH and NPM‐naSAH is poorly studied. Smoking and hypertension could be a risk factor for PM‐naSAH, but the results are inconsistent (Mensing et al., 2018). In our study, smoking or hypertension was not a risk factor for PM‐naSAH. One possible reason for this is that we compared risk factors between PM‐naSAH and NPM‐naSAH, while earlier studies has compared PM‐naSAH to general population or aneurysmal SAH (Mensing et al., 2018).

Similar to our findings, in earlier case series PM‐naSAH has occurred in 40%–77% of patients after physical exertion, but unlike our study, these studies did not compare differences between PM‐naSAH and NPM‐naSAH (Linn et al., 1998; Matsuyama et al., 2006). The source of bleeding in naSAH is unknown, but venous, arterial, or capillary source of bleeding has been proposed (Rouchaud et al., 2016). Recent study found that PM‐naSAH patients had more variants in basal vein of Rosenthal compared to NPM‐naSAH patients, suggesting that PM‐naSAH could be from venous origin (Fang et al., 2021). One theory is that Valsalva maneuver during the physical exertion could potentially cause venous hypertension which leads to bleeding (Rouchaud et al., 2016). In contrast to PM‐naSAH, we found that NPM‐naSAH occurred only in 16% of patients after physical exertion, which is almost identical to aneurysmal SAH (Anderson et al., 2003; Vlak et al., 2011).

Based on a different bleeding pattern (Cánovas et al., 2012; Kang et al., 2009; Konczalla et al., 2016;Raya et al., 2014), clinical course (Cánovas et al., 2012 ;Kang et al., 2009; Konczalla et al., 2016 ; Raya et al., 2014), and triggering events according to our findings, it is possible that PM‐naSAH and NPM‐naSAH have different pathophysiological background.

### 4.1 Limitations

This was a retrospectively study and triggering event for PM‐naSAH and NPM‐naSAH was based on the mentions in the patient records. However, we were able to achieve accurate triggering events in 89% of included patients, most likely because most of the patients with naSAH is in a good clinical condition at the admission. One of the strength of this study was that we reviewed all patients who had undergone digital subtraction angiography at our tertiary hospital; as a result, we were probably able to find all naSAH patients with negative digital subtraction angiography. Our tertiary hospital is responsible of all nontraumatic SAH patients and diagnosis in our catchment area of 890 000 citizens, which minimize the selection bias in the register based studies.

## 5 CONCLUSION

Physical exertion was a common trigger for PM‐naSAH and the risk was five times higher compared to NPM‐naSAH. One explanation for this finding could be that PM‐naSAH and NPM‐naSAH has a different pathophysiological background. More studies are needed to confirm our results and to study pathophysiology of PM‐naSAH and NPM‐naSAH.

## FINANCIAL DISCLOSURE

Authors have indicated they have no financial relationships relevant to this article to disclose.

## CONFLICT OF INTEREST

Authors have indicated they have no potential conflicts of interest to disclose. Clinical Trial Registration: None.

### PEER REVIEW

The peer review history for this article is available at: https://publons.com/publon/10.1002/brb3.2756.

## Data Availability

The datasets generated during and/or analyzed during the current study are available from the corresponding author on reasonable request.

## References

[brb32756-bib-0001] 1 Anderson, C. , Ni Mhurchu, C. , Scott, D. , Bennett, D. , Jamrozik, K. , Hankey, G. , & Australasian Cooperative Research on Subarachnoid Hemorrhage Study Group . (2003). Triggers of subarachnoid hemorrhage: Role of physical exertion, smoking, and alcohol in the Australasian Cooperative Research on Subarachnoid Hemorrhage Study (ACROSS). Stroke, 34(7), 1771–1776. 10.1161/01.STR.0000077015.90334.A7 12775890

[brb32756-bib-0002] 2 Cánovas, D. , Gil, A. , Jato, M. , de Miquel, M. , & Rubio, F. (2012). Clinical outcome of spontaneous non‐aneurysmal subarachnoid hemorrhage in 108 patients. European Journal of Neurology, 19(3), 457–461. 10.1111/j.1468-1331.2011.03542.x 21972883

[brb32756-bib-0003] 3 Fang, Y. , Shao, A. , Wang, X. , Lu, J. , Wu, H. , Ren, R. , Huang, Y. i. , Lenahan, C. , Xu, J. , Chen, S. , & Zhang, J. (2021). Deep venous drainage variant rate and degree may be higher in patients with perimesencephalic than in non‐perimesencephalic angiogram‐negative subarachnoid hemorrhage. European Radiology, 31(3), 1290–1299. 10.1007/s00330-020-07242-5 32918092

[brb32756-bib-0004] 4 Fann, J. R. , Kukull, W. A. , Katon, W. J. , & Longstreth, W. T. Jr. (2000). Physical activity and subarachnoid haemorrhage: A population based case‐control study. Journal of Neurology, Neurosurgery, and Psychiatry, 69(6), 768–772. 10.1136/jnnp.69.6.768 11080229 PMC1737186

[brb32756-bib-0005] 5 Kang, D. H. , Park, J. , Lee, S. H. , Park, S.‐H. , Kim, Y.‐S. , & Hamm, I.‐S. (2009). Does non‐perimesencephalic type non‐aneurysmal subarachnoid hemorrhage have a benign prognosis? Journal of Clinical Neuroscience, 16(7), 904–908. 10.1016/j.jocn.2008.10.008 19362482

[brb32756-bib-0006] 6 Konczalla, J. , Kashefiolasl, S. , Brawanski, N. , Senft, C. , Seifert, V. , & Platz, J. (2016). Increasing numbers of nonaneurysmal subarachnoid hemorrhage in the last 15 years: Antithrombotic medication as reason and prognostic factor? Journal of Neurosurgery, 124(6), 1731–1737. 10.3171/2015.5.JNS15161 26566212

[brb32756-bib-0007] 7 Linn, F. H. , Rinkel, G. J. , Algra, A. , & van Gijn, J. (1998). Headache characteristics in subarachnoid haemorrhage and benign thunderclap headache. Journal of Neurology, Neurosurgery, and Psychiatry, 65(5), 791–793. 10.1136/jnnp.65.5.791 9810961 PMC2170334

[brb32756-bib-0008] 8 Matsuyama, T. , Okuchi, K. , Seki, T. , Higuchi, T. , & Murao, Y. (2006). Perimesencephalic nonaneurysmal subarachnoid hemorrhage caused by physical exertion. Neurologia Medico‐Chirurgica, 46(6), 277–281. discussion 281–2. 10.2176/nmc.46.277 16794347

[brb32756-bib-0009] 9 Mensing, L. A. , Vergouwen, M. D. I. , Laban, K. G. , Ruigrok, Y. M. , Velthuis, B. K. , Algra, A. , & Rinkel, G. J. E (2018). Perimesencephalic hemorrhage: A review of epidemiology, risk factors, presumed cause, clinical course, and outcome. Stroke, 49(6), 1363–1370. 10.1161/STROKEAHA.117.019843 29695465

[brb32756-bib-0010] 10 Raya, A. , Zipfel, G. J. , Diringer, M. N. , Dacey, R. G. Jr , Derdeyn, C. P. , Rich, K. M. , Chicoine, M. R. , & Dhar, R. (2014). Pattern not volume of bleeding predicts angiographic vasospasm in nonaneurysmal subarachnoid hemorrhage. Stroke, 45(1), 265–267. 10.1161/STROKEAHA.113.002629 24193803

[brb32756-bib-0011] 11 Rinkel, G. J. , Wijdicks, E. F. , Hasan, D. , Vermeulen, M. , & Hageman, L. M (1991). Outcome in patients with subarachnoid haemorrhage and negative angiography according to pattern of haemorrhage on computed tomography. Lancet, 338(8773), 964–968. 10.1016/0140-6736(91)91836-j 1681340

[brb32756-bib-0012] 12 Rinkel, G. J. , Wijdicks, E. F. , Vermeulen, M. , Ramos, L. M. P. , anghe, H. L. J. T. , Hasan, D. , Meiners, L. C. , & Gijn, J. v. (1991). Nonaneurysmal perimesencephalic subarachnoid hemorrhage: CT and MR patterns that differ from aneurysmal rupture. AJNR American Journal of Neuroradiology, 12(5), 829–834. PMID: 1950905; PMCID: PMC8333493. 1950905 PMC8333493

[brb32756-bib-0013] 13 Rouchaud, A. , Lehman, V. T. , Murad, M. H. , Burrows, A. , Cloft, H. J. , Lindell, E. P. , Kallmes, D. F. , & Brinjikji, W. (2016). Nonaneurysmal perimesencephalic hemorrhage is associated with deep cerebral venous drainage anomalies: A systematic literature review and meta‐analysis. AJNR American Journal of Neuroradiology, 37(9), 1657–1663. 10.3174/ajnr.A4806 27173362 PMC7984705

[brb32756-bib-0014] 14 Schievink, W. I. , Karemaker, J. M. , Hageman, L. M. , & van der Werf, D. J. (1989). Circumstances surrounding aneurysmal subarachnoid hemorrhage. Surgical Neurology, 32(4), 266–272. 10.1016/0090-3019(89)90228-0 2675363

[brb32756-bib-0015] 15 Vlak, M. H. , Rinkel, G. J. , Greebe, P. , van der Bom, J. G. , & Algra, A. (2011). Trigger factors and their attributable risk for rupture of intracranial aneurysms: A case‐crossover study. Stroke; A Journal of Cerebral Circulation , 42(7), 1878–1882. 10.1161/STROKEAHA.110.606558 21546472

